# Torsion of a bifid omentum as a rare cause of acute abdomen: a case report

**DOI:** 10.1186/s13256-016-1070-9

**Published:** 2016-10-19

**Authors:** Vicky Dhooghe, David Reynders, Peter Cools

**Affiliations:** Department of Abdominal Surgery, GZA Hospitals, Campus Sint Vincentius, Sint Vincentiusstraat 20, 2018 Antwerp, Belgium

**Keywords:** Omental torsion, Acute abdomen, Surgical resection

## Abstract

**Background:**

Omental torsion is a rare and very unusual cause of acute abdominal pain. If often mimics other acute pathologies and it is very difficult to diagnose preoperatively, which can lead to deterioration of the patient. It is seldom reported in the literature.

**Case presentation:**

We report a well-documented case of a 67-year-old white woman who complained about abdominal pain, which was slowly increasing in severity. She had no previous abdominal interventions. An abdominal ultrasound showed multiple gallstones. At laparoscopy, free hemorrhagic fluid was seen and further exploration showed torsion of the right part of her omentum. A partial omentectomy was performed. Her postoperative course was uneventful.

**Conclusions:**

Omental torsion is a rare cause of abdominal pain. Primary omental torsion is seldom reported in the literature. Blood examinations are frequently normal. Abdominal ultrasound and computed tomography can exclude other pathologies. Exploration remains the preferred diagnostic and therapeutic modality. Surgeons should include the diagnosis of omental torsion in their differential diagnosis of acute abdominal pain.

## Background

Omental torsion and infarction are rare and unusual causes of acute abdominal pain. Omental torsion and infarction is caused by the twisting of the omentum along its long axis compromising its vascularity. It often mimics other acute pathologies and is very difficult to diagnose preoperatively, which can lead to the deterioration of the patient. We report a case of primary omental torsion with infarction of the right part of a bifid greater omentum.

## Case presentation

A 67-year-old obese white woman consulted the gastroenterologist in our hospital with a 2-day history of abdominal pain located in her right hypochonder, which was slowly increasing in severity. She did not complain of symptoms such as nausea, vomiting, or diarrhea. She had no history of abdominal problems.

**Fig. 1:**
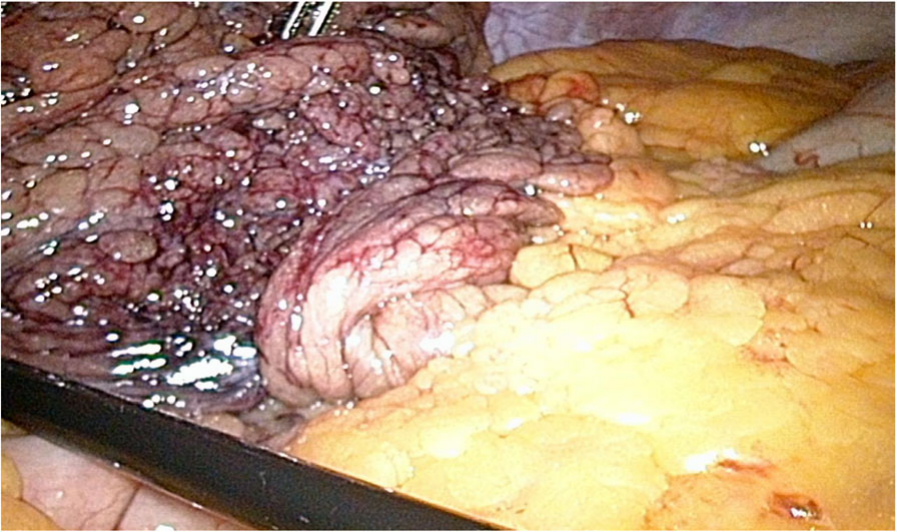
Intraoperative view of the congested, necrotic omentum

A clinical examination revealed a tender right hemiabdomen with percussion pain. Laboratory tests demonstrated leukocytosis (10.2 × 1000/mm^3^), normal liver function test, and an elevated C-reactive proteine (CRP) (43.4 mg/L). An abdominal ultrasound showed multiple gallstones with a normal choledochal duct, lacking significant signs of acute cholecystitis. Because of the presumption of symptomatic cholecystolithiasis, our patient underwent a laparoscopic exploration. Inspection of her peritoneal cavity revealed free intra-abdominal hemorrhagic fluid. Further exploration showed torsion of the right part of her omentum, which can be visualized in Fig. 1.

**Fig. 2:**
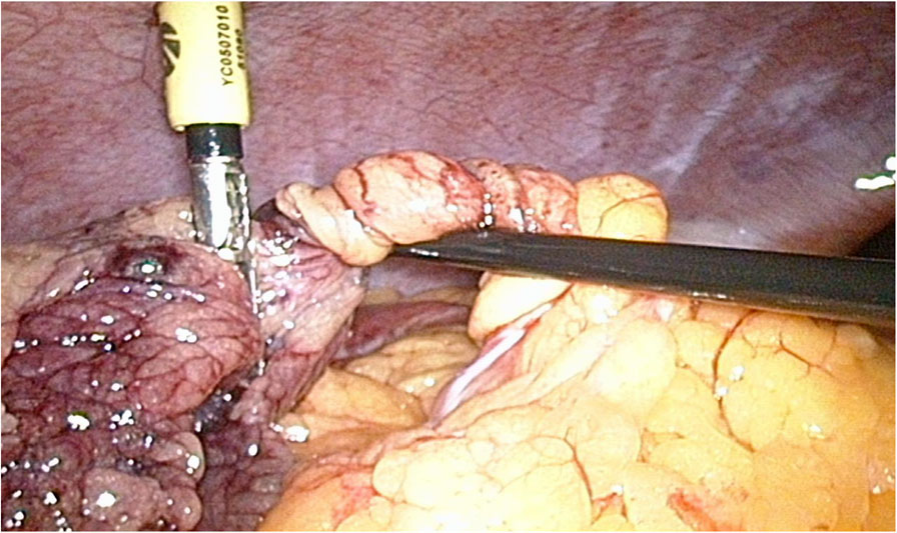
Intraoperative view of the twisted vascular axis

**Fig. 3:**
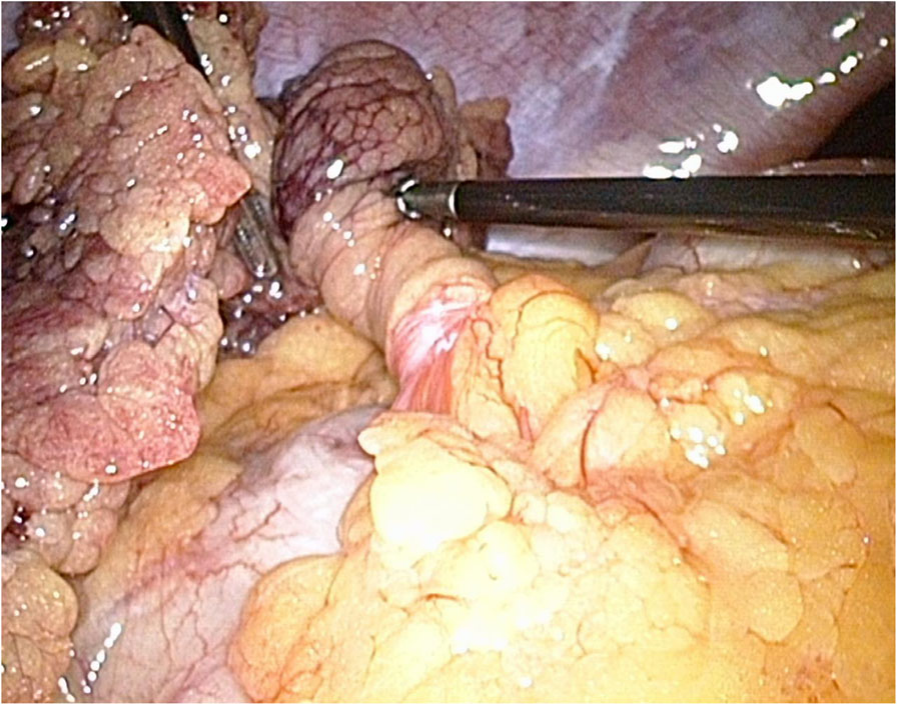
The right part of the bifid omentum is twisted; the left part is normal

**Fig. 4:**
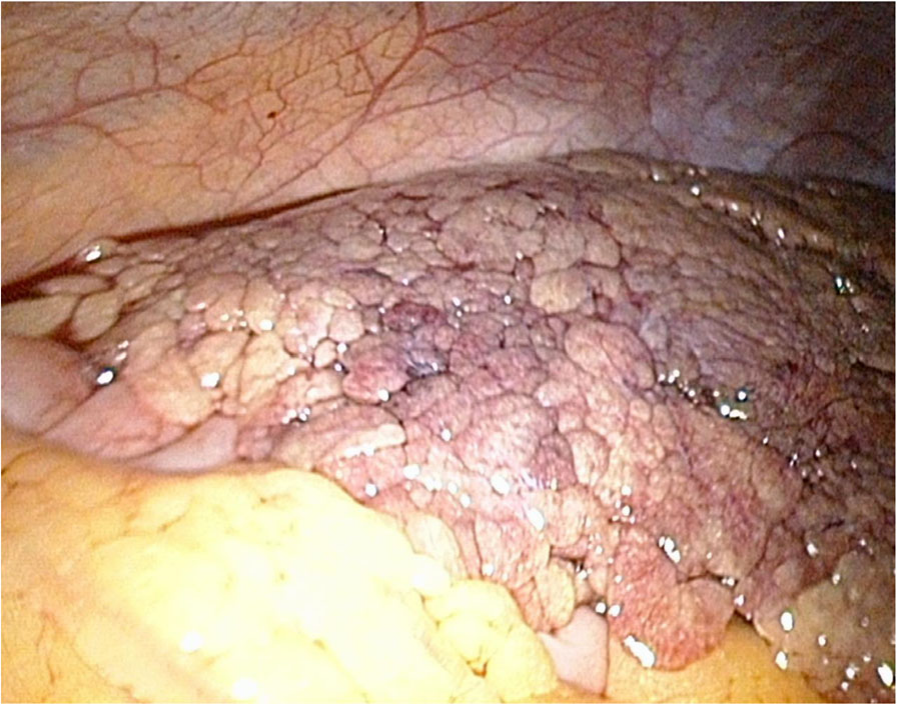
The omentum is partially necrotic

**Fig. 5:**
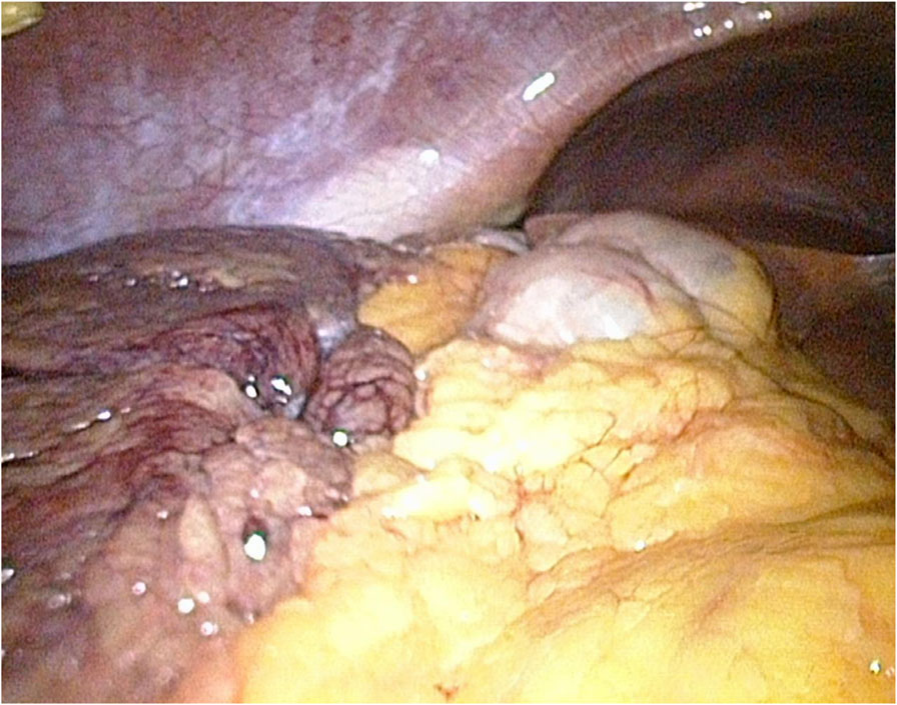
Partially necrotic and partially healthy omentum are seen

Her bifid omentum was twisted around its vascular axis several times, as demonstrated in Figs. 2 and 3. Because of the necrotic aspect of her omentum, shown in Figs. 4 and 5, a partial omentectomy was performed. Her postoperative course was uneventful and she could be discharged from our hospital after 2 days.

## Discussion

Omental torsion is a rare cause of acute abdominal pain, which can present in two ways. Eitel first described primary omental torsion in 1899 [1]. Anatomical malformations, such as a bifid or accessory omentum consisting of an abnormal embryological position of the right part of the omentum with secondary fragile vascularity and abnormal deposits of fat, are predisposed for omental torsion [2, 3]. The omentum twists around a pivotal point impairing its vascular perfusion resulting in congestion and edema [4, 5].

Omental torsion mainly affects adults; it affects males twice as frequently as females, with the majority being overweight [6]. Reports have described its prevalence in children [2, 6, 7]. Omental displacement caused by trauma, violent exercise, hyperperistalsis, or compression between the abdominal wall and liver are precipitating factors, but its primary cause remains unknown [2–4]. Secondary omental torsion is more common and is associated with predisposing pathologies such as intra-abdominal inflammation, adhesions, tumors, or cysts. The dependent omentum is fixed in a torsed position and unable to untwist [3]. Detortion has been described but is very rare [8]. Without detortion, arterial occlusion leads to acute hemorrhagic infarction and necrosis of the omentum will occur.

The primary symptom associated with omental torsion is pain, which is frequently localized in the right part of the abdomen [3]. The pain has an acute onset and does not radiate to the abdominal wall [9]. It can mimic other causes of acute abdomen such as appendicitis, cholecystitis, and diverticulitis; in women it can mimic gynecologic diseases [10]. Therefore, omental torsion should be included in the differential diagnosis of acute abdomen.

Blood examinations are frequently found to be normal. Because of the clinical context of an acute abdomen, ultrasound and computed tomography are useful to assist the diagnosis. Classical signs of omental torsion on computed tomography are the whirl sign of a fatty mass with concentric linear strands [11]. Computed tomography can also exclude other pathologies such as acute appendicitis, cholecystitis, and diverticulitis. Omental infarction is only diagnosed preoperatively in 4.8 % of cases because of the nonspecific clinical symptoms [3, 12].

Exploration remains the preferred diagnostic and therapeutic modality [5, 10]. Surgical management of primary omental torsion includes resection of the involved omentum. Early diagnosis may lead to conservative management, although surgery has been recommended for avoiding severe complications such as sepsis and intra-abdominal abscess formation [13].

## Conclusions

Omental torsion is an unusual cause of acute abdominal pain with nonspecific symptoms and signs of acute abdomen, making diagnosis very difficult. Surgeons should include it in their differential diagnosis of acute abdomen. Computed tomography can be useful to reveal the diagnosis or to exclude other pathologies. Surgical resection of the infarcted omentum remains the treatment of choice.
